# Top 100 Publications as Measured by Altmetrics in the Field of Central Nervous System Inflammatory Demyelinating Disease

**DOI:** 10.1155/2019/3748091

**Published:** 2019-12-02

**Authors:** Jee-Eun Kim, Yerim Kim, Kang Min Park, Dae Young Yoon, Jong Seok Bae

**Affiliations:** ^1^Department of Neurology, Seoul Medical Center, Seoul, Republic of Korea; ^2^Department of Neurology, Kangdong Sacred Heart Hospital, Hallym University College of Medicine, Seoul, Republic of Korea; ^3^Department of Neurology, Haeundae Paik Hospital, Inje University College of Medicine, Busan, Republic of Korea; ^4^Department of Radiology, Kangdong Sacred Heart Hospital, Hallym University College of Medicine, Seoul, Republic of Korea

## Abstract

**Background:**

Altmetrics analyze the visibility of articles in social media and estimate their impact on the general population. We performed an altmetric analysis of articles on central nervous system inflammatory demyelinating disease (CIDD) and investigated its correlation with citation analysis.

**Methods:**

Articles in the 91 journals comprising the “clinical neurology,” “neuroscience,” and “medicine, general, and internal” Web of Science categories were searched for their relevance to the CIDD topic. The Altmetric Explorer database was used to determine the Altmetric.com Attention Score (AAS) values of the selected articles. The papers with the top 100 AAS values were characterized.

**Results:**

Articles most frequently mentioned online were primarily published after 2014 and were published in journals with high impact factors. All articles except one were dealt with the issue of multiple sclerosis. Most were original articles, but editorials were also common. Novel treatments and risk factors are the most frequent topics. The AAS was weakly correlated with journal impact factors; however, no link was found between the AAS and the number of citations.

**Conclusions:**

We present the top 100 most frequently mentioned CIDD articles in online media using an altmetric approach. Altmetrics can rapidly offer alternative information on the impact of research based on a broader audience and can complement traditional metrics.

## 1. Introduction

Traditionally, the impact of each scientific article is indirectly measured by its citation count or the impact factor of the journal publishing it. The impact factor is calculated as the average citation number of the articles that were published in that journal during the preceding two years (number of total citations/number of total citable articles). However, accessibility to websites is dramatically increasing worldwide, and the number of social media users has reached almost 2.46 billion in 2017 [1]. One-third of people worldwide are anticipated to use social media in 2021 [1]. Because of these shifts, the channels and audiences for science communication are inexorably increasing. Altmetrics, also called alternative metrics, capture the online visibility of scholarly material and indirectly present its influence on social networks [2]. Altmetrics quantify the frequency of mentions in online channels including news outlets, science blogs, Twitter, Facebook, Sina Weibo, Wikipedia, public policy documents, peer review platforms, research highlights on Faculty of 1000, reference manager such as Mendeley, and multimedia (YouTube and Q&A) [3]. The Altmetric.com Attention Score (AAS), one of the commonly used altmetric tools, is calculated depending on the intensity, types (e.g., retweets are scored lower than original tweets), and sources of attention (e.g., articles shared through news outlets are weighted 8, but those shared on Twitter are weighted 1) [3]. A high AAS, as calculated by this method, represents noteworthy interest in an article within social media outlets [4]. Altmetrics have certain advantages over traditional metrics: the former can measure the effect of scientific works outside the academic community and can estimate the impact of discoveries more quickly than citation analysis [4].

Multiple sclerosis (MS), the most representative form of central nervous system inflammatory demyelinating disease (CIDD), has been an important issue in the health care community because of its relatively high prevalence, chronicity, and massive social burden. MS typically develops in young adults aged 20–50 years, and its incidence peaks in the fourth decade of life [5]. These young adult patients are especially familiar with Internet use, and they actively acquire and spread information on the disease by social media or other digital outlets [6]. There are numerous official and unofficial social networks, web pages, and blogs on the topic of MS [7].

Here, our purpose is to identify the most influential articles in the field of CIDD in online media and to investigate the characteristics of those articles. We hypothesized that traditional metrics and altmetrics were not necessarily related. We compared the ASS to a traditional citation analysis to find the relationship between the two metrics.

## 2. Materials and Methods

### 2.1. Article Selection and Analysis

We searched journals in the categories of “clinical neurology,” “neuroscience,” and “medicine, general, and internal” in the 2016 edition of Web of Science (Thomson Reuters, New York, NY) to further identify the CIDD-related articles that were most commonly mentioned in digital media. A total of 91 journals were selected, and articles published in those journals were subjected to further analysis. We used the AAS as a weighted measure of the attention received by each article in digital media [3]. The AAS of articles in selected journals were searched separately in the Altmetric Explorer database (Altmetric LLP, London, UK) on May 25, 2018. All articles were compiled as a single database and rearranged in order of descending AAS. To retrieve CIDD-related articles, we chose articles with the following terms in their titles: “multiple sclerosis,” “clinically isolated syndrome,” “neuromyelitis optica,” “neuromyelitis optica spectrum disorder (NMOSD),” “optic neuritis,” “myelitis,” “demyelinating disease,” “Devic's disease,” “aquaporin-4 antibody,” “neuromyelitis optica-immunoglobulin G,” “Schilder's disease,” “Schilder's diffuse sclerosis,” “diffuse myelinoclastic sclerosis,” “Balo concentric sclerosis,” “Marburg multiple sclerosis,” “solitary sclerosis,” “acute disseminated encephalomyelitis,” or “acute hemorrhagic leukoencephalitis.” After reviewing the original texts to confirm their relevance to CIDD, we identified the top 100 articles by AAS. To avoid the risk of overestimating public attention because of single articles dealing with many different diseases, we excluded articles that dealt with multiple diseases (e.g., an article that evaluated the effect of living near a major road on the risk of multiple sclerosis, dementia, and Parkinson's disease had the highest AAS in the present study, but it was excluded from further evaluation) [8]. Articles were reviewed for the selection process regardless of language, scholarly identifier, or document type.

These mixed methods study utilized quantitative analyses, qualitative assessment, and an extensive review of literature. The following information was extracted from the top 100 most highly mentioned CIDD-related articles in online media: article title, year of publication (if electronic publishing was opened ahead of formal publication, the year of electronic publishing was selected for further analysis), published journal, journal impact factor, country of origin, type of article, main topics, subject of article (e.g., MS, NMOSD, and myelitis), AAS, and number of citations. Country of origin was defined by the affiliation of the first author. If the first author had more than one affiliation, then the affiliation of the corresponding author was used to determine the country of origin. The journal impact factor was extracted from the 2017 edition of Web of Science. The traditional citation count for each article was obtained from Scopus and compared to the AAS.

The study was exempted from approval by an ethics committee because it was a bibliometric analysis.

### 2.2. Statistical Analysis

All statics were analyzed using SPSS Statistics 20 (SPSS, Chicago, IL, USA). Pearson's correlation analysis was performed to assess the relationship between AAS and journal impact factor (or citation count). *p* < 0.05 was regarded as statistically significant.

## 3. Results

**Figure 1:**
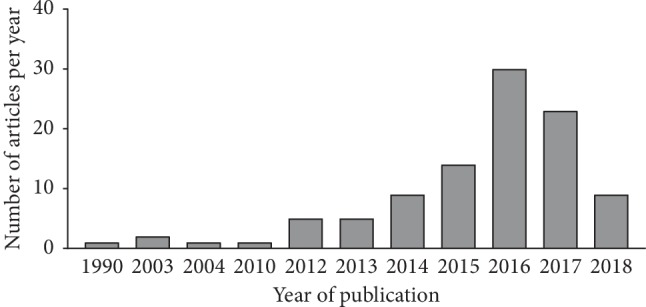
Articles with the top 100 highest Altmetric.com Attention Scores, sorted by the year of publication.

We identified 100 articles with the highest altmetric scores (Table 1). AAS values were in the range of 113 to 1302 (median 214.5). The median journal impact factor of the articles with the top 100 altmetric scores was 7.690 (range 2.45–79.26). The median number of citations per article was 21 (range 0–765). Altmetric.com has tracked attention paid to articles mentioned in each source since 2011. The identified articles were published between 1990 and 2018, and 85% of the articles were published after 2014 (Figure 1). Articles on the top 100 list originated from 15 different countries (Table 2). Approximately 60% of the top 100 articles originated in Northern America. The remaining high-ranked countries of origin were on the European continent. Interestingly, the top 100 articles discussed online were published mostly in journals with high impact factors (Table 3). Among 23 journals, *Neurology* (*n* *=* *31*) has the most articles ranked in the top 100, followed in order, by the *Journal of the American Medical Association Neurology* (*n* *=* *14*), *The Lancet* (*n* *=* *11*), the *Journal of Neurology, Neurosurgery and Psychiatry* (*n* *=* *10*), and *The New England Journal of Medicine* (*n* *=* *8*). Except for one article explaining clinical biomarkers to differentiate inflammatory myelopathy from other causes, the subject of all the articles was MS. No articles on the subject of NMOSD were in the top 100. However, two NMOSD articles had notably high AAS values. One article suggesting 2015 NMOSD diagnostic criteria ranked 112 (AAS = 98), and an article regarding the clinical course and therapeutic efficiency of NMOSD ranked 125 (AAS = 88) [9, 10].

Original articles were the most frequent type among the top 100 highly mentioned CIDD papers, and 60% of the original articles were clinical observational studies (Table 4). Approximately 10% of highly mentioned articles were reviews, meta-analyses, or articles suggesting practical guidelines or diagnostic criteria. Editorial articles were also included in the list of high-ranking publications (Table 4). Most of these editorial articles summarized notable recent studies and provided expert opinions regarding new treatments (including B-cell depletion treatments such as ocrelizumab and alemtuzumab, BCG vaccines, hematopoietic stem-cell transplantation, and percutaneous transluminal venous angioplasty) and risk factors (such as coffee consumption, chronic cerebrospinal venous insufficiency, diet, vitamin D, and smoking). The main issues addressed by the top 100 articles were mostly related to disease treatment, and many of them were comparative clinical trials (Table 5). The specific treatments receiving the most attention are presented in Table 6. One article was a retrospective cohort study presenting the complication of malignancy after mitoxantrone treatment in MS. The second most frequent class of topics consisted of the risk factors for MS development and aggravation (Table 6). Notably, several high-ranking articles concerned quality-of-life or economic issues.

**Figure 2:**
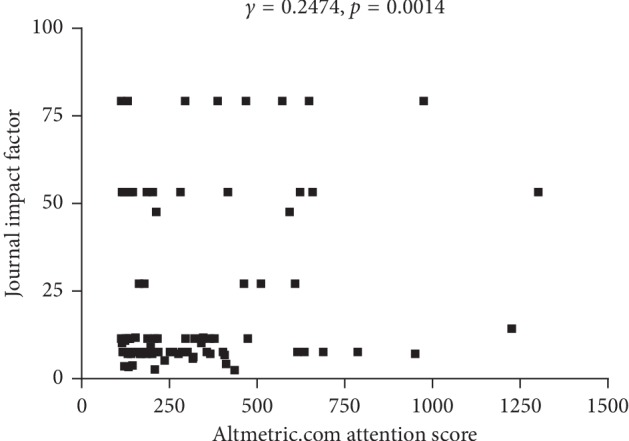
Correlation between Altmetric.com Attention Scores and journal impact factors among the top 100 articles on central nervous system inflammatory demyelinating disease mentioned most online.

Regarding the correlation between AAS and journal impact factor among the top 100 articles, a weak positive correlation was revealed (*r* = 0.2474; *p*=0.0014) (Figure 2). However, there was no significant correlation between AAS and citation counts.

## 4. Discussion

We performed an altmetric analysis of CIDD articles to examine their influences on not only academia but also the general public. We thus obtained a list of the 100 articles in the field of CIDD that were most frequently mentioned in web media according to weighted AAS. These results show trends in scientific research and social attention on a shorter time lag than traditional citation analysis. Among our top 100 articles, AAS showed correlation with journal impact factor but not with citation count, which means that altmetrics are correlated with traditional metrics to some extent but do not convey identical information.

Traditional citation analysis has a long history as a tool for measuring the impact of an article, authors, and contributed institutes/countries. Citation analysis offers a “best-seller list” of the specific diseases that receive the most attention in academia. However, this form of analysis has several limitations. In practice, citations of an article start 1–2 years after its initial publication and peak between 3 and 10 years [11]. After considerable periods, some important articles cease to be frequently cited because their substance has been absorbed into the current body of common knowledge, the phenomenon called “obliteration by incorporation” [12]. For this reason, traditional analysis best captures the actual impact of a paper 10 to 20 years after publication [13, 14]. Altmetrics are a new class of metrics that can indicate the impact of an article in a more rapid and responsive way than citation analysis. Previous results comparing altmetrics and citation analysis have shown that new web-based metrics are especially sensitive to the latest news [15, 16]. Reflecting this characteristic, most of the top 100 CIDD articles in our study were published after 2014, and a plurality of articles were published in 2016. Furthermore, when we considered the relationship between citation number and AAS, we could not find any correlation. A substantial number of articles on the top 100 list were cited fewer than 10 times, which might be due to the scope of the audience, the document types, and the time since publication. Our results support the idea that citation analysis and altmetrics do not measure the same construct; they are complementary rather than substitutable. The absence of a strong correlation between new metrics and citation analysis was consistently observed in other studies [17–23]. Only Mendeley, an online reference manager, showed a moderate positive link with citation analysis [19, 20].

In our correlation analysis, AAS showed a weak positive correlation with a journal impact factor. In addition, the top 5 most represented journals, which included 74% of the listed articles, have impact factors of more than 7. This result may have occurred because articles published in higher impact journals are generally more interesting in their own right or because of the fame and wide reach of these journals. Several factors are known to influence AAS, such as journal impact factor, article length, number of collaborators or references, presence of a press release, free accessibility, document type, and funding [16, 24]. Several factors, such as reference count and collaborative practices, positively affect both citations and altmetrics [16]. However, the impacts of other factors are not identical [16]. Longer papers typically receive more citations, but the opposite trend can be seen on social media platforms [16]. Editorial and news documents, which are rarely cited, are frequently mentioned document types on Twitter [16]. Simple declarative titles that present key conclusions are associated with high AAS values [25–27]. Our study also showed substantial inclusion of editorial articles, which distill topics to their essentials, and are easier to understand than research papers. Brevity and understandability are important factors in the dissemination of articles on websites [25].

The most frequently mentioned topics were associated with novel therapeutics and risk factors (especially correctable environmental factors). Articles concerning disease mechanisms, clinical characteristics, and diagnosis also accounted for a portion of the list. However, compared with traditional citation analysis, altmetrics show increased attention to treatment and decreased attention to diagnosis and disease mechanisms [28]. Similarly, articles that address socioeconomic and quality-of-life issues of CIDD patients are also highly ranked in terms of altmetrics, an outcome that is seldom observed in citation analysis [28]. Other researchers also reported that research papers relating to humanities and social science are most frequently found on online media, in contrast to the pattern in conventional media [16]. Our findings indirectly represent the difference between audience and user groups (academic-based versus a more inclusive public audience) and their interest in conventional and social media. The top 100 articles mostly originated in North America and Europe. These results may have occurred because many core studies were performed in those areas. Another influence might be the large volume of attention paid to MS in Northern America and Europe because of its relatively high prevalence and because of widespread Internet use [1, 5].

Several aspects of this study should be considered. First, altmetrics are new methods of bibliometrics, and their value was uncertain until now. Several inherent limitations of altmetrics have been discussed recently [4, 21, 29]. Social media are at risk of overestimating the impact of research with sensational outcomes [21, 29]. Articles with negative findings or attractive headlines are likely to have high AAS values [21]. Some scientists have reported that metrics from online platforms do not accurately reflect the quality research [30]. AAS scores cannot distinguish the depth of online mentions although the algorithm is adjusted to weight scores by the source and type of attention [22]. Furthermore, due to the anonymity of social media, the demographics of the users referencing the articles (scientists or the lay public) and the validity of the data are often not certain [21, 29]. As in traditional citation analysis, altmetrics may be influenced by temporal bias [12]. Second, we used only altmetric data offered by Altmetric.com in this study. Social media metrics in each data aggregator can differ due to methodological (e.g., selections on the data collection) and reporting choices (e.g., data aggregation approaches) and have both advantages and disadvantages [31]. Other tools such as ALS-PLoS, ImpactStory, and Plum Analytics can also be used to track altmetrics data. Third, we did not perform subgroup analyses based on the attention source subtype. Further studies focusing on difference in the social media subset are expected.

## 5. Conclusion

We identified the 100 research outputs most discussed on online platforms shared by a broad audience of scientists and laypeople. There was some discordance between traditional citation analysis and altmetrics analysis of CIDD articles. Data from altmetrics may not accurately represent scientific quality or importance, but they can reflect the dissemination of research through the general population, including the scientific community, on the web. The influence of online media will inevitably increase in the future. Altmetrics can provide a broad, real-time web-based reflection of research and can complement traditional analysis as a tool to evaluate the impact of studies. Further research will be needed to assess the validity of altmetrics and explore the factors that influence them.

## Figures and Tables

**Table 1:** List of the top 100 articles by Altmetric.com Attention Scores.

Rank	Article	First author	IF	Altmetric.com Attention Score	No. of citations
1	Immunoablation and autologous haemopoietic stem-cell transplantation for aggressive multiple sclerosis: a multicentre single-group phase 2 trial, Lancet, 2016, 388, 576.	Atkins H. L.	53.254	1302	68
2	Nuclear receptor NR1H3 in familial multiple sclerosis, Neuron, 201692, 555.	Wang Z.	14.318	1226	24
3	Ocrelizumab versus placebo in primary progressive multiple sclerosis, N Engl J Med, 2017, 376, 209.	Mantalban X.	79.258	975	220
4	Time to wake up and smell the coffee? Coffee consumption and multiple sclerosis, J Neurol Neurosurg Psychiatry, 2016, 87, 453.	Wijnands J. M.	7.144	951	1
5	Breastfeeding, ovulatory years, and risk of multiple sclerosis, Neurology, 2017, 89, 563.	Langer-Gould A.	7.609	787	5
6	Alemtuzumab improves preexisting disability in active relapsing-remitting multiple sclerosis patients, Neurology, 2016, 87, 1985.	Giovannoni G.	7.609	689	17
7	Siponimod versus placebo in secondary progressive multiple sclerosis (EXPAND): a double-blind, randomised, phase 3 study, Lancet, 2018, 391, 1263.	Kappos L.	53.254	659	16
8	A placebo-controlled trial of oral cladribine for relapsing multiple sclerosis, N Engl J Med, 2010, 362, 416.	Giovannoni G.	79.258	649	332
9	Pediatric multiple sclerosis, Neurology, 2016, 87, S74.	Waldman A.	7.609	635	15
10	Alemtuzumab for patients with relapsing multiple sclerosis after disease-modifying therapy: a randomised controlled phase 3 trial, Lancet, 2012, 380, 1829.	Coles A. J.	53.254	623	506
11	Superior MRI outcomes with alemtuzumab compared with subcutaneous interferon *β*-1a in multiple sclerosis, Neurology, 2016, 87, 1464.	Arnold D. L.	7.609	615	6
12	Effect of oral cladribine on time to conversion to clinically definite multiple sclerosis in patients with a first demyelinating event (ORACLE MS): a phase 3 randomised trial, Lancet Neurol, 2014, 13, 257.	Leist T. P.	27.138	609	50
13	Quadrivalent HPV vaccination and risk of multiple sclerosis and other demyelinating diseases of the central nervous system, JAMA, 2015, 313, 54.	Scheller N. M.	47.661	593	76
14	Multiple Sclerosis, N Engl J Med, 2018, 378, 169.	Reich D. S.	79.258	572	31
15	Safety and efficacy of the selective sphingosine 1-phosphate receptor modulator ozanimod in relapsing multiple sclerosis (RADIANCE): A randomised, placebo-controlled, phase 2 trial, Lancet Neurol, 2016, 15, 373.	Cohen J. A.	27.138	511	38
16	Long-term outcomes after autologous hematopoietic stem cell transplantation for Multiple sclerosis, JAMA Neurol, 2017, 74, 459.	Muraro P. A.	11.46	474	30
17	Ocrelizumab versus interferon beta-1a in relapsing multiple sclerosis, N Engl J Med, 2016, 376, 221.	Hauser S. L.	79.258	469	167
18	Estriol combined with glatiramer acetate for women with relapsing-remitting multiple sclerosis: a randomised, placebo-controlled, phase 2 trial, Lancet Neurol, 2016, 15, 35.	Voskuhl R. R.	27.138	463	49
19	Predisposing role of vitamin D receptor (VDR) polymorphisms in the development of multiple sclerosis: a case-control study, J Neurol Sci, 2016, 367, 148.	Abdollahzadeh R.	2.448	436	7
20	Direct and indirect cost burden associated with multiple sclerosis relapses: excess costs of persons with multiple sclerosis and their spouse caregivers, J Neurol Sci, 2013, 330, 71.	Parisé H.	2.448	435	20
21	Effect of high-dose simvastatin on brain atrophy and disability in secondary progressive multiple sclerosis (MS-STAT): A randomised, placebo-controlled, phase 2 trial, Lancet, 2014, 383, 2213.	Chataway J.	53.254	417	162
22	Targeting demyelination and virtual hypoxia with high-dose biotin as a treatment for progressive multiple sclerosis, Neuropharmacology, 2015, 110, 644.	Sedel F.	4.249	412	44
23	Anti-spasticity agents for multiple sclerosis, Cochrane database Sys Rev, 2003, (4), CD001332.	Shakespeare D.	6.754	408	137
24	The cost of multiple sclerosis drugs in the US and the pharmaceutical industry: too big to fail?, Neurology, 2015, 84, 2185.	Hartung D. M.	7.609	403	84
25	Trial of minocycline in a clinically isolated syndrome of multiple sclerosis, N Engl J Med, 2017, 376, 2122.	Metz L. M.	79.258	388	23
26	Efficacy and safety of extracranial vein angioplasty in multiple sclerosis: a randomized clinical trial, JAMA Neurol, 2017, 75, 35.	Zamboni P.	11.46	379	8
27	High consumption of coffee is associated with decreased multiple sclerosis risk; results from two independent studies, J Neurol Neurosurg, Psychiatry, 2016, 87, 454.	Hedström A. K.	7.144	367	18
28	Vitamin D status during pregnancy and risk of multiple sclerosis in offspring of women in the Finnish maternity cohort, JAMA Neurol, 2016, 73, 515.	Munger K. L.	11.46	366	41
29	Diet quality is associated with disability and symptom severity in multiple sclerosis, Neurology, 2018, 90, e1.	Fitzgerald K. C.	7.609	357	5
30	Effects of physical comorbidities on disability progression in multiple sclerosis, Neurology, 2018, 90, e419.	Zhang T.	7.609	355	3
31	Vitamin D and risk of multiple sclerosis: A Mendelian randomization study, PLoS Med, 2015, 12, e1001866.	Mokry LE	11.675	347	110
32	Vaccines and the risk of multiple sclerosis and other central nervous system demyelinating diseases, JAMA Neurol, 2014, 71, 1506.	Langer-Gould A.	11.46	344	58
33	Concussion in adolescence and risk of multiple sclerosis, Ann Neurol, 2017, 82, 554.	Montgomery S.	10.244	341	6
34	High-dose immunosuppressive therapy and autologous hematopoietic cell transplantation for relapsing-remitting multiple sclerosis (HALT-MS): A 3-year interim report, JAMA Neurol, 2015, 72, 159.	Nash R. A.	11.46	322	73
35	Smoked cannabis for spasticity in multiple sclerosis: A randomised, placebo-controlled trial, CMAJ, 2012, 184, 1143.	Corey-Bloom J.	6.21	319	84
36	Cladribine to treat relapsing forms of multiple sclerosis, Neurotherapeutics, 2017, 14, 874.	Giovannoni G.	5.719	317	2
37	Safety and immunologic effects of high- vs low-dose cholecalciferol in multiple sclerosis, Neurology, 2016, 86, 382.	Sotirchos E. S.	7.609	302	45
38	Neonatal vitamin D status and risk of multiple sclerosis A population-based case-control study, Neurology, 2017, 88, 44.	Nielsen N. M.	7.609	297	24
39	Association of nonmyeloablative hematopoietic stem cell transplantation with neurological disability in patients with relapsing-remitting multiple sclerosis, JAMA Neurol, 2015, 313, 275.	Burt R. K.	11.46	296	66
40	Daclizumab HYP versus Interferon Beta-1a in Relapsing Multiple Sclerosis, N Engl J Med, 2015, 373, 1418.	Kappos L.	79.258	295	132
41	25-Hydroxyvitamin D deficiency and risk of multiple sclerosis among women in the Finnish Maternity cohort, Neurology, 2017, 89, 1578.	Munger K. L.	7.609	292	5
42	Sun exposure over the life course and associations with multiple sclerosis, Neurology, 2018, 90, e1191.	Tremlett H.	7.609	286	—^a^
43	Clemastine fumarate as a remyelinating therapy for multiple sclerosis (ReBUILD): a randomised, controlled, double-blind, crossover trial, Lancet, 2017, 90, 2481.	Green A. J.	53.254	282	34
44	Higher latitude is significantly associated with an earlier age of disease onset in multiple sclerosis, J Neurol Neurosurg Psychiatry, 2016, 87, 1343.	Tao C.	7.144	276	10
45	No association between dietary sodium intake and the risk of multiple sclerosis, Neurology, 2017, 89, 1322.	Cortese M.	7.609	260	7
46	Practice guideline recommendations summary: disease-modifying therapies for adults with multiple sclerosis: report of the guideline development, dissemination, and implementation subcommittee of the American Academy of neurology, Neurology, 2018, 90, 777.	Rae-Grant A.	7.609	252	—^a^
47	Rab32 connects ER stress to mitochondrial defects in multiple sclerosis, J Neuroinflammation, 2017, 14, 19.	Haile Y.	5.193	237	8
48	High-dose immunosuppressive therapy and autologous hematopoietic cell transplantation for relapsing-remitting multiple sclerosis, Neurology, 2017, 88, 842.	Nash R. A.	7.609	219	21
49	Comprehensive systematic review summary: disease-modifying therapies for adults with multiple sclerosis: report of the guideline development, dissemination, and implementation subcommittee of the American Academy of neurology, Neurology, 2018, 90, 789.	Rae-Grant A.	7.609	217	—^a^
50	Vitamin D as an early predictor of multiple sclerosis activity and progression, JAMA Neurol, 2014, 71, 306.	Ascherio A.	11.46	216	203
51a	New inroads against multiple sclerosis, JAMA, 2018, 319, 9.	Lyon J.	47.661	213	2
51b	Neurofilament light chain level is a weak risk factor for the development of multiple sclerosis, Neurology, 2016, 87, 1076.	Arrambide G.	7.609	213	20
53	Price analysis of multiple sclerosis disease-modifying therapies marketed in the United States, Curr Med Res Opin, 2016, 32, 1783.	Bin Sawad A.	2.665	209	3
54	Combating the spread of ineffective medical procedures: A lesson learned from multiple sclerosis, JAMA Neurol, 2017, 75, 15.	Green A. J.	11.46	208	0
55	Clinical biomarkers differentiate myelitis from vascular and other causes of myelopathy, Neurology, 2017, 90, e12.	Barreras P.	7.609	206	2
56	Prevalence of extracranial venous narrowing on catheter venography in people with multiple sclerosis, their siblings, and unrelated healthy controls: a blinded, case-control study, Lancet, 2014, 383, 138.	Traboulsee A. L.	53.254	203	44
57	Role of genetic susceptibility variants in predicting clinical course in multiple sclerosis: a cohort study, J Neurol Neurosurg Psychiatry, 2016, 87, 1204.	Pan G.	7.144	201	12
58	Myalgic encephalomyelitis/chronic fatigue syndrome and encephalomyelitis disseminata/multiple sclerosis show remarkable levels of similarity in phenomenology and neuroimmune characteristics, BMC Med, 2013, 11, 205.	Morris G.	9.088	197	54
59a	Effect of smoking cessation on multiple sclerosis prognosis, JAMA Neurol, 2015, 72, 1117.	Ramanujam R.	11.46	188	49
59b	Physical activity and the incidence of multiple sclerosis, Neurology, 2016, 87, 1770.	Dorans K.	7.609	188	3
61a	Chronic cerebrospinal venous insufficiency in multiple sclerosis: the final curtain, Lancet, 2014, 383, 106.	Paul F.	53.254	186	5
61b	Diet and disease modification in multiple sclerosis: A nutritional epidemiology perspective, J Neurol Neurosurg Psychiatry, 2018, 89, 3.	Fitzgerald K.	7.144	186	—^a^
63	Diagnosis of multiple sclerosis: 2017 revisions of the McDonald criteria, Lancet Neurol, 2018, 17, 162.	Thompson A. J.	27.138	179	88
64	Assessing response to interferon-*β* in a multicenter dataset of patients with multiple sclerosis, Neurology, 2016, 87, 134.	Sormani M. P.	7.609	177	28
65a	Disconnection as a mechanism for social cognition impairment in multiple sclerosis, Neurology, 2017, 89, 38.	Batista S.	7.609	170	7
65b	Helicobacter pylori infection as a protective factor against multiple sclerosis risk in females, J Neurol Neurosurg Psychiatry, 2015, 86, 603.	Pedrini M. J.	7.144	170	21
67	HIV and lower risk of multiple sclerosis: beginning to unravel a mystery using a record-linked database study, J Neurol Neurosurg Psychiatry, 2015, 86, 9.	Gold J.	7.144	167	38
68	Health-care use before a first demyelinating event suggestive of a multiple sclerosis prodrome: a matched cohort study, Lancet Neurol, 2017, 16, 445.	Wijnands J. M. A.	27.138	164	14
69	Autologous hematopoietic stem cell transplantation in multiple sclerosis: a phase II trial, Neurology, 2015, 84, 981.	Mancardi G. L.	7.609	162	79
70	Psychiatric comorbidity is associated with disability progression in multiple sclerosis, Neurology, 2018, 90, e1316.	McKay K. A.	7.609	158	2
71	Obesity and multiple sclerosis: a Mendelian randomization study, PLoS Med, 2016, 13, e1002053.	Mokry L. E.	11.675	154	35
72	BCG vaccine for clinically isolated syndrome and multiple sclerosis: infections and protective immunity, Neurology, 2013, 82, 15.	Bourdette D.	7.609	152	5
73	Efficacy of balance and eye-movement exercises for persons with multiple sclerosis (BEEMS), Neurology, 2018, 90, e797.	Hebert J. R.	7.609	148	—^a^
74	Cannabinoids for treatment of spasticity and other symptoms related to multiple sclerosis (CAMS study): multicentre randomised placebo-controlled trial, Lancet, 2003, 362, 1517.	Zajicek J.	53.254	146	528
75	Taste dysfunction in multiple sclerosis, J Neurol, 2016, 263, 677.	Doty R. L.	3.783	145	5
76	Association between age at onset of multiple sclerosis and vitamin D level-related factors, Neurology, 2016, 86, 88.	Laursen J. H.	7.609	144	7
77	Multiple sclerosis and extract of cannabis: results of the MUSEC trial, J Neurol Neurosurg Psychiatry, 2012, 83, 1125.	Zajicek J. P.	7.144	141	95
78a	Haemopoietic stem-cell transplantation for multiple sclerosis: what next?, Lancet, 2016, 388, 536.	Dörr J.	53.254	139	2
78b	Sodium intake is associated with increased disease activity in multiple sclerosis, J Neurol Neurosurg Psychiatry, 2015, 86, 26.	Farez M. F.	7.144	139	92
80a	Exclusive breastfeeding and the effect on postpartum multiple sclerosis relapses, JAMA Neurol, 2015, 72, 1132.	Hellwig K.	11.46	138	30
80b	Malignancies after mitoxantrone for multiple sclerosis A retrospective cohort study, Neurology, 2016, 86, 2203.	Martinelli V.	7.609	138	16
82	Noninvasive tongue stimulation combined with intensive cognitive and physical rehabilitation induces neuroplastic changes in patients with multiple sclerosis: a multimodal neuroimaging study, Mult Scler J Exp Transl Clin, 2017, 3, 2055217317690561.	Leonard G.	—	137	3
83	Comparative effectiveness of rituximab and other initial treatment choices for multiple sclerosis, JAMA Neurol, 2018, 75, 320.	Granqvist M.	11.46	136	7
84	Hormone therapy use and physical quality of life in postmenopausal women with multiple sclerosis, Neurology, 2016, 87, 1457.	Bove R.	7.609	135	5
85a	Summary of evidence-based guideline: complementary and alternative medicine in multiple sclerosis report of the guideline development Subcommittee of the American Academy of neurology, Neurology, 2014, 82, 1083.	Yadav V.	7.609	134	64
85b	The underdiagnosis of sleep disorders in patients with multiple sclerosis, J Clin Sleep Med, 2014, 10, 1025.	Brass S. D.	3.396	134	41
86b	Selection of patients with multiple sclerosis to undergo autologous hematopoietic stem cell transplantation, JAMA Neurol, 2017, 74, 392.	Racke M. K.	11.46	132	0
88	Contribution of dietary intake to relapse rate in early pediatric multiple sclerosis, J Neurol Neurosurg Psychiatry, 2018, 89, 28.	Azary S.	7.144	132	5
89	B-cell depletion-a frontier in monoclonal antibodies for multiple sclerosis, N Engl J Med, 2017, 376, 280.	Calabresi P. A.	79.258	131	7
90	Vitamin D during pregnancy and multiple sclerosis: an evolving association, JAMA Neurol, 2016, 73, 498.	Greenberg B. M.	11.46	125	4
91	Neuroendothelial NMDA receptors as therapeutic targets in experimental autoimmune encephalomyelitis, Brain, 2016, 139, 2406.	Macrez R.	10.84	124	5
92	Pain in people with multiple sclerosis: associations with modifiable lifestyle factors, fatigue, depression, anxiety, and mental health quality of life, Front Neurol, 2017, 8, 461.	Marck C. H.	3.508	122	0
93a	Effect of low saturated fat diet in early and late cases of multiple sclerosis, Lancet, 1990, 336, 37.	Swank R. L.	53.254	121	120
93b	The contemporary spectrum of multiple sclerosis misdiagnosis: a multicenter study, Neurology, 2016, 87, 1393.	Solomon A. J.	7.609	121	40
95	Sustained reduction of multiple sclerosis disability: new player in comparing disease-modifying treatments, Neurology, 2016, 87, 1966.	Bielekova B.	7.609	119	1
96	Recombinant hepatitis B vaccine and the risk of multiple sclerosis: a prospective study, Neurology, 2004, 63, 838.	Hernán M. A.	7.609	117	202
97a	Alemtuzumab versus interferon beta 1a as first-line treatment for patients with relapsing-remitting multiple sclerosis: a randomised controlled phase 3 trial, Lancet, 2012, 380, 1819.	Cohen J. A.	53.254	115	516
97b	Genes and environment in multiple sclerosis project: a platform to investigate multiple sclerosis risk, Ann Neurol, 2016, 79, 178.	Xia Z.	10.244	115	14
99a	Placebo-controlled phase 3 study of oral BG-12 for relapsing multiple sclerosis, N Engl J Med, 2012, 367, 1098.	Gold R.	79.258	113	765
99b	Smoking beyond multiple sclerosis diagnosis: a risk factor still worth modifying, JAMA Neurol, 2015, 72, 1105.	Goldman M. D.	11.46	113	0

IF: impact factor; No.: number. ^a^The number of citations was not obtainable at the time of investigation in recently published articles in Scopus database.

**Table 2:** Countries of origin of articles highly mentioned online.

	Number of top 100 publications
*Countries of origin*	
United States of America	40
Canada	18
United Kingdom	11
Australia	5
Germany	5
Sweden	5
Italy	3
Spain	3
Denmark	2
France	2
Switzerland	2
Argentina	1
Iran	1
Norway	1
Portugal	1

**Table 3:** Journals where the central nervous system inflammatory demyelinating disease articles with the highest Altmetric.com Attention Scores were published.

Rank	Journal	Number of top 100 publications
1	Neurology	31
2	Journal of the American Medical Association Neurology	14
3	The Lancet	11
4	Journal of Neurology, Neurosurgery and Psychiatry	10
5	The New England Journal of Medicine	8
6	The Lancet Neurology	5
7	Annals of Neurology	2
7	Journal of the American Medical Association	2
7	Journal of the Neurological Sciences	2
7	PLOS Medicine	2
8	Brain	1
8	BMC Medicine	1
8	Canadian Medical Association Journal	1
8	Cochrane Database Systematic Review	1
8	Current Medical Research and Opinion	1
8	Frontiers in Neurology	1
8	Journal of Clinical Sleep Medicine	1
8	Journal of Neuroinflammation	1
8	Journal of Neurology	1
8	Neuron	1
8	Neuropharmacology	1
8	Neurotherapeutics	1
8	Multiple Sclerosis Journal-Experimental Translational and Clinical	1

**Table 4:** Types of articles with top 100 Altmetric.com Attention Scores.

Category	No. of articles
Original article	
Comparative clinical trial	26
Clinical observational study	50
Basic research	3
Review/meta-analysis	6
Diagnostic criteria/guidelines	3
Editorial	12

**Table 5:** General issues discussed in the highly mentioned articles on central nervous system inflammatory demyelinating disease.

Category	No. of articles
Treatment and related complications	45
Risk factors	
Environmental factors	31
Genetic factors	5^a^
CSF biomarkers	1
Diagnosis and clinical characteristics	8
Disease mechanism	5
Economic and quality-of-life issues	5

CSF: cerebrospinal fluid; No.: number. ^a^The Genes and Environment in Multiple Sclerosis (GEMS) project in the United States evaluated both genetic and environmental factors that lead to multiple sclerosis development in at-risk people; publication based on the GEMS was arbitrarily counted as articles on the subject of genetic risk factor.

**Table 6:** Topics on treatment and risk factor of central nervous system inflammatory demyelinating disease that achieved high attention in social outlets.

Category	Specific topic
Treatment	Haemopoietic stem cell transplantation
	B-cell depleting agents (ocrelizumab and alemtuzumab)
	Siponimod
	Oral cladribine
	Sphingosine 1-phosphate receptor modulator ozanimod
	Estriol combined with glatiramer acetate
	High-dose simvastatin
	High-dose biotin
	Cannabis and other antispastic agents
	Minocycline
	Extracranial vein angioplasty
	Cholecalciferol
	Daclizumab high-yield process
	Clemastine fumarate
	Interferon-*β*
	Oral BG-12
	Balance and eye-movement exercises
	Noninvasive tongue stimulation with intensive cognitive/physical rehabilitation

Risk factors	*Modifiable lifestyle*: coffee consumption, breastfeeding, ovulatory years, vaccination, vitamin D, sun exposure, diet (sodium intake and low saturated fat diet), concussion, latitude, physical comorbidities, exercise, smoking, Helicobacter pylori infection, HIV infection
	*Genetic factors*: nuclear receptor NR1H3, vitamin D receptor polymorphisms, single nucleotide polymorphisms associated with MS risk, the results of the genes and environment in multiple sclerosis (GEMS) project in the United States

## Data Availability

The data used to support the findings of this study are available from the corresponding author upon request.

## Abbreviations

AAS: Altmetric.com Attention Score
CIDD: Central nervous system inflammatory demyelinating disease
CSF: Cerebrospinal fluid
IF: Impact factor
MS: Multiple sclerosis
NMOSD: Neuromyelitis optica spectrum disorder
No: Number.

## References

[1] The Statistics Portal (2018). *Social Media Statistics & Facts*.

[2] Chavda J., Patel A. (2016). Measuring research impact: bibliometrics, social media, altmetrics, and the BJGP. *British Journal of General Practice*.

[3] Altmetric Support (2018). How is the altmetric attention score calculated?. https://help.altmetric.com/support/solutions/articles/6000060969-how-is-the-altmetric-attention-score-calculated-.

[4] Trueger N. S., Thoma B., Hsu C. H., Sullivan D., Peters L., Lin M. (2015). The altmetric score: a new measure for article-level dissemination and impact. *Annals of Emergency Medicine*.

[5] Milo R., Kahana E. (2010). Multiple sclerosis: geoepidemiology, genetics and the environment. *Autoimmunity Reviews*.

[6] Smith A., Anderson M., Pew Research Center (2018). *Social Media Use in 2018*.

[7] Lavorgna L., Brigo F., Moccia M. (2018). e-Health and multiple sclerosis: an update. *Multiple Sclerosis Journal*.

[8] Chen H., Kwong J. C., Copes R. (2017). Living near major roads and the incidence of dementia, Parkinson’s disease, and multiple sclerosis: a population-based cohort study. *The Lancet*.

[9] Wingerchuk D. M., Banwell B., Bennett J. L. (2015). International consensus diagnostic criteria for neuromyelitis optica spectrum disorders. *Neurology*.

[10] Kleiter I., Gahlen A., Borisow N. (2016). Neuromyelitis optica: evaluation of 871 attacks and 1,153 treatment courses. *Annals of Neurology*.

[11] Marx W., Schier H., Wanitschek M. (2001). Citation analysis using online database: feasibilities and shortcomings. *Scientometrics*.

[12] Garfield E. (1987). 100 citation classics from the journal of the American medical association. *JAMA*.

[13] Albert D. M. (1988). Analysis of the Archives’ most frequently cited articles. *Archives of Ophthalmology*.

[14] Hall G. M. (1998). BJA citation classics 1945–1992. *British Journal of Anaesthesia*.

[15] Araújo R., Sorensen A. A., Konkiel S., Bloem B. R. (2017). Top altmetric scores in the Parkinson’s disease literature. *Journal of Parkinson’s Disease*.

[16] Haustein S., Costas R., Larivière V. (2015). Characterizing social media metrics of scholarly papers: the effect of document properties and collaboration patterns. *PLoS One*.

[17] Thelwall M., Haustein S., Larivière V., Sugimoto C. R. (2013). Do Altmetrics work? Twitter and ten other social web services. *PLoS One*.

[18] Barbic D., Tubman M., Lam H., Barbic S. (2016). An analysis of altmetrics in emergency medicine. *Academic Emergency Medicine*.

[19] Haustein S., Larivière V., Thelwall M. (2014). Tweets vs. Mendeley readers: how do these two social media metrics differ?. *Information Technology*.

[20] Amath A., Ambacher K., Leddy J. J., Wood T. J., Ramnanan C. J. (2017). Comparing alternative and traditional dissemination metrics in medical education. *Medical Education*.

[21] O’Connor E. M., Nason G. J., O’Kelly F., Manecksha R. P., Loeb S. (2017). Newsworthiness vs scientific impact: are the most highly cited urology papers the most widely disseminated in the media?. *BJU International*.

[22] Delli K., Livas C., Spijkervet F. K. L., Vissink A. (2017). Measuring the social impact of dental research: an insight into the most influential articles on the web. *Oral Diseases*.

[23] Barakat A. F., Nimri N., Shokr M. (2019). Correlation of altmetric attention score and citations for high-impact general medicine journals: a cross-sectional study. *Journal of General Internal Medicine*.

[24] Haneef R., Ravaud P., Baron G. (2017). Factors associated with online media attention to research: a cohort study of articles evaluating cancer treatments. *Research Integrity and Peer Review*.

[25] Di Girolamo N., Reynders R. M. (2017). Health care articles with simple and declarative titles were more likely to be in the altmetric top 100. *Journal of Clinical Epidemiology*.

[26] Costas R., Zahedi Z., Wouters P. (2015). Do “Altmetrics” correlate with citations? extensive comparison of altmetric indicators with citations from a multidisciplinary perspective. *Journal of the Association for Information Science and Technology*.

[27] Peters I., Kraker P., Lex E., Gumpenberger C., Gorraiz J. (2016). Research data explored: an extended analysis of citations and altmetrics. *Scientometrics*.

[28] Kim J.-E., Park K. M., Kim Y., Yoon D. Y., Bae J. S. (2017). Citation classics in central nervous system inflammatory demyelinating disease. *Brain and Behavior*.

[29] Warren H. R., Raison N., Dasgupta P. (2017). The rise of altmetrics. *JAMA*.

[30] Bornmann L., Haunschild R. (2018). Do altmetrics correlate with the quality of papers? a large-scale empirical study based on F1000Prime data. *PLoS One*.

[31] Zahedi Z., Costas R. (2018). General discussion of data quality challenges in social media metrics: extensive comparison of four major altmetric data aggregators. *PLoS One*.

